# Improving medication adherence in stroke survivors: the intervention development process

**DOI:** 10.1186/s12913-018-3572-1

**Published:** 2018-10-11

**Authors:** Elise Crayton, Alison J Wright, Mark Ashworth

**Affiliations:** 1 Faculty of Life Sciences and Medicine, School of Population Health & Environmental Sciences, King’s College London, London, UK; 2 Centre for Behaviour Change, University College London, 1-19 Torrington Place, London, UK

**Keywords:** Stroke, Medication adherence, Health behavior, Secondary prevention, Intervention development

## Abstract

**Background:**

Medications targeting stroke risk factors have shown good efficacy, yet adherence is suboptimal. A lack of underlying theory may contribute to the ineffectiveness of eliciting or sustaining behaviour change in many existing interventions targeting medication adherence in stroke. Intervention effectiveness and implementation could be enhanced by consideration of evidence base and theory to drive development. The purpose of this study is to identify appropriate components for a theory-driven and evidence-based medication adherence intervention for stroke survivors.

**Methods:**

The Behaviour Change Wheel (BCW), a guide to intervention development, informed our systematic process of intervention development. Our earlier systematic review had identified important determinants of medication adherence that were mapped into the Theoretical Domains Framework (TDF), with Knowledge, Beliefs about consequences and Emotions found to be more influential. Utilising the BCW facilitated selection of intervention options and behaviour change techniques (BCTs); the active ingredients within an intervention. To further refine BCT selection, APEASE criteria were employed, allowing evaluation of potential BCTs within context: The National Health Service (NHS), United Kingdom (UK).

**Results:**

Five intervention functions (Education, Persuasion, Training, Environmental Restructuring and Enablement) and five policy categories (Communication/marketing, Guidelines, Regulation, Environmental/social planning and Service provision) were identified as potential intervention options, underpinned by our systematic review findings. Application of APEASE criteria led to an initial pool of 21 BCTs being reduced to 11 (e.g. Habit Formation, Information about Health Consequences and Action Planning) identified as potential intervention components that would both be feasible and directly target the underlying determinants of stroke survivors’ medication adherence.

**Conclusions:**

Careful consideration of underlying evidence and theory to drive intervention design, facilitated by the BCW, enabled identification of appropriate intervention components. BCTs including Habit Formation, Information about Health Consequences and Self-monitoring of Behaviour were considered potentially effective and appropriate to deliver within the NHS. Having reduced the pool of potential intervention components to a manageable number, it will now be possible to explore the perceived acceptability of selected BCTs in interviews with stroke survivors and healthcare professionals. This approach to intervention development should be generalisable to other chronic conditions and areas of behaviour change (e.g. exercise adherence).

## Background

Stroke can result in life altering and fatal consequences [1]. In 2013, stroke was recognised as the second leading cause of death worldwide [2], with an estimated 110,000 first or recurrent stroke in the United Kingdom (UK) per annum [3]. Moreover, cumulative risk of a secondary stroke is thought to be as much as 26% in the first five years following a stroke [4]. Guidelines recommend medications for the secondary prevention of stroke [3, 5]), including antihypertensive, blood thinning and cholesterol lowering medications.

It is evident that medication adherence, defined as “the extent to which the patient’s action matches the agreed recommendations” [6], is sub-optimal in stroke survivors (e.g. [6, 7]), despite the efficacy of medications for risk factor control of cardiovascular conditions such as hypertension and hyperlipidaemia [8–10], with about a third of stroke survivors considered non-adherent [11]. Many attempts have been made to intervene, in an effort to improve medication adherence. Unfortunately, the majority of interventions targeting medication adherence in stroke survivors have shown limited effectiveness [12, 13]. This has also been the case in other chronic conditions [14]. The complexity of measuring adherence and the variability in reasons identified for non-adherence are reflected in results from previous intervention studies [12, 13]. This limits the ability to know whether the intervention components are in fact targeting true influences of medication adherence and whether adherence is being accurately measured. The UK Medical Research Council (MRC) framework for designing and evaluating complex interventions has advocated systematic intervention development, using evidence base and theory, [15] and other frameworks for intervention development also suggest similar, for example Intervention Mapping [16, 17]. A lack of evidence-based selection of behaviour change techniques (BCTs, the active ingredients within an intervention [18]) used in the intervention may be partially responsible for limited success to date. Therefore, this study aimed to develop an evidence-based and theory driven behaviour change intervention targeting medication adherence in stroke survivors.

There are numerous theories of behaviour that can underpin intervention development [19]. However, these theories have been subject to a number of criticisms, including not always operationalising the constructs clearly, not considering the context in which a behaviour occurs, and an over emphasis on rational, deliberative determinants [20]. The development of the Theoretical Domains Framework (TDF) offers some response to these criticisms, and provides a more holistic model of behaviour to underpin intervention development [21, 22]. The TDF was developed through expert consensus. Behaviour change professionals identified constructs from many major behaviour change theories. The identified constructs were clustered using open and closed sort tasks, grouping similar constructs together to form, what the authors termed, a domain. After revisions, 14 key domains were established (Knowledge; Skills; Social/Professional role and identity; Beliefs about capabilities; Optimism; Beliefs about consequences; Reinforcement; Intentions; Goals; Memory, Attention and Decision processes; Environmental context and resources; Social influences; Emotions; & Behavioural regulation [22]).

The Behaviour Change Wheel (BCW) is a guide to intervention development that has provided a systematic and structured intervention development process, underpinned by theory: the TDF [22, 23]. This has enhanced intervention development processes, such that researchers are able to make evidence-based selection of intervention components (including BCTs, the intervention’s active ingredients), ensuring that interventions target the underlying determinants of behaviour. The BCW prescribes a process of systematically mapping underlying determinants of behaviour, in a series of stages, to BCTs that are perceived to best target and influence these determinants. Moreover, BCTs have been refined into a taxonomy of 93 BCTs [18], providing a consistent language to use and a resource to access a comprehensive list of BCTs when developing interventions. Research applying the BCW to underpin intervention design, in other health behaviours (such as safer sex), have shown good feasibility and acceptability of the interventions, with emerging evidence to support intervention effectiveness in influencing outcomes [24].

Application of literature, consideration of the context in which the intervention will be delivered and use of APEASE (Affordability, Practicality, Effectiveness, Acceptability, Side effects, Equity; [25]) evaluative criteria will enhance the intervention development, outlined in this study. Systematic reviews can assist in identifying the evidence base for the determinants of the behaviour. This provides foundations for later evidence-based selection of intervention components most likely to elicit behaviour change. The evidence base for this intervention development was drawn from a recent systematic review, identifying psychological determinants of medication adherence in stroke survivors [26]. The determinants identified were mapped into the domains of the TDF, as this was the theoretical framework selected to underpin the intervention development. The series of steps advocated by the BCW to develop an intervention, which align with other guidance on developing complex interventions [15], were utilised and are described in detail in the methods below. The methods also discuss how literature, consideration of the context the intervention will be delivered in and use of evaluative criteria were utilised to ensure that components selected for the intervention were not only targeting the underlying determinants of behaviour, but were also appropriate and realistic to be delivered within the desired context, in this case within the UK National Health Service (NHS). The aim of this study was to identify appropriate components for a theory-driven and evidence-based medication adherence intervention for stroke survivors.

## Methods

**Fig. 1:**
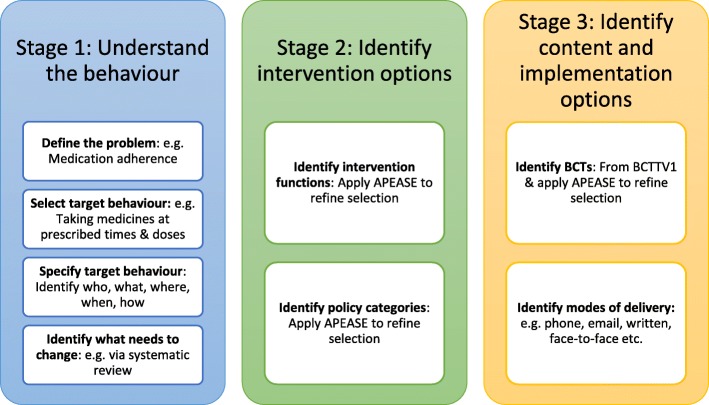
Illustrating a systematic and theory driven intervention development process. BCTTV1 – Behaviour Change Technique Taxonomy Version 1. APEASE - Affordability, practicality, effectiveness/cost-effectiveness, acceptability, side effects, equity

The authors followed a systematic process of intervention development advocated by the BCW guidance [25], which involved a series of stages that are discussed below and outlined in Fig. 1. This process was underpinned by previous research [26], that formed the initial steps of this intervention development. Any intervention development decision was based on the premise that this intervention would be suitable for all adult stroke survivors, except those living in long-term institutional care or those that are fully reliant on carers for all daily tasks, as medication adherence would be largely under the control of a carer.

Each stage followed will be discussed in more detail below.

### Stage 1: Understanding the behaviour

This stage comprises four steps (detailed in Fig. 1) that facilitate defining the public health problem in context. This was approached by reviewing evidence to define the behavioural problem (Step 1), breaking this down into specific behaviours and eventually selecting one target behaviour to focus upon (Step 2 and 3). A systematic review of psychological determinants of medication adherence in stroke survivors [26] supported understanding of influences upon the behaviour (Step 4). Mapping the identified determinants into the TDF and identifying key domains that were more influential to behaviour change enabled a better understanding of what needed to change.

### Stage 2: Identifying intervention options

This stage of intervention development was supported by the Behaviour Change Wheel guidance [23, 25], encompassing two steps. This drew on previous research, whereby a systematic review identified 19 frameworks of behaviour change that encompassed the nine intervention functions and seven policy categories used in the BCW [23]. Moreover, a consensus exercise undertaken by experts in the field, linked theoretical domains to intervention functions and subsequent policy categories well suited to facilitate the behaviour change [23, 25]. Intervention functions are defined as “broad categories of means by which an intervention can change behaviour” [25] and policy categories can be understood as “types of decisions made by authorities that help to support and enact the intervention” [25]. Use of this literature facilitated identification of relevant intervention functions (Step 1) and policy categories (Step 2). Extensive work has been carried out to identify the intervention functions and policy categories most likely to bring about change, when linked to underlying theoretical domains form the TDF [25]. A consideration of the likely services (e.g. health, voluntary, social etc.) that the intervention could be implemented into also facilitated choice of intervention functions and realistic policy categories. Application of APEASE [25] provided evaluative criteria and also narrowed down the potential intervention functions that would be carried forward for this intervention development.

Whilst effectiveness of an intervention is certainly important to consider during the design process, there are other factors that are important, such as evaluation of intervention component suitability in the intended setting and social context. APEASE evaluative criteria support this broader consideration by challenging an intervention designer to ask questions such as [25]:

- (Affordability) Is the cost of the proposed intervention within budget?
- (Practicality) Can the intervention be delivered as designed in the intended setting?
- (Effectiveness) How effective is the intervention? What is the magnitude (effect size) of the relationship between intervention and behaviour?
- (Acceptability) Is the intervention deemed appropriate by key stakeholders?
- (Side effects) Are there any unwanted side effects from delivering this intervention that need to be considered?
- (Equity) Does the intervention reduce or increase disparities between different sectors of society?

Therefore, the use of APEASE criteria supplements the selection of intervention components through the application of contextual criteria.

### Stage 3: Identify content and implementation options

An emerging literature base from experts in the field, identifying BCTs better suited to certain intervention functions and underlying theoretical domains, supported evidence-based selection of appropriate BCTs [27, 28]. In addition, extensive literature searching of current BCTs used in interventions deemed to be effective, targeting akin behaviours or patient groups, as well as application of APEASE criteria supported BCT choice. Affordability, practicality and acceptability were deemed to be of particular importance in this evaluative process by the research team, given the NHS context for implementation. To set the scene, the NHS provides universal health coverage and it is a service in high demand, in part because it is free at the point of use. At present, the UK norm for length of a general practitioner (GP) consultation is a 10-min appointment, although some practices are able to offer longer appointments. Nurse appointments in primary care are often longer. Community pharmacists do not usually operate an appointment system but offer consultations based on clinical requirements. HCPs such as community pharmacists are increasingly enlisted to deliver interventions to enhance healthcare. For example, pharmacists offer medication use reviews (MURs) annually to patients and this may offer an alternative to GP care. Specific to stroke services, patients discharged from a secondary care setting will often only have one or two follow up appointments with the secondary care team before being discharged to primary care services, where the secondary prevention of stroke through medication use, for example, will then be managed. BCTs found not to meet these three APEASE criteria were not carried forward to the next stage of intervention design.

## Results

### Stage 1: Understanding the behaviour

The target behaviour for this intervention is medication adherence, defined as “the extent to which the patient’s action matches the agreed recommendations” [6]. As discussed earlier, up to a third of stroke survivors are non-adherent to their medications, indicating the behavioural problem for this intervention (Step 1).

The target behaviour for this intervention was further defined as: stroke survivors taking medication at the prescribed times, doses and frequencies (Step 2 and 3). A decision was made, following consultation with a patient and public involvement group, to target all medication as opposed to narrowing the focus down to one medication, such as an antihypertensive. A systematic review identifying the psychological factors that influence stroke survivors’ medication adherence was already available [26] (Step 4). Determinants including *concerns about medications*, *beliefs about medication necessity*, *knowledge about medications* and *negative emotions* had been identified as influential [26]. Once mapped into the TDF, three key domains were identified as influential in behaviour change: ‘Knowledge’ *(knowledge about medications)*, ‘Beliefs about consequences’ *(concerns about medications*, *beliefs about medication necessity)* and ‘Emotions’ *(negative emotions)* domains.

### Stage 2: Identifying intervention options

A mapping process, recommended by BCW guidance [23, 25] was followed. This drew on previous research [23, 25], utilised to identify the potential intervention functions and policy categories that would target the three theoretical domains found to be most influential to medication adherence in stroke survivors in Stage 1. The possible intervention functions (Step 1) and policy categories (Step 2) are displayed in Tables 1 and 2.

**Table 1:** Intervention functions that are appropriate to target underlying theoretical domains

TDF Domain	Intervention Function	Intervention Function Definitions^a^	Included/excluded from next stage	Reasons for Inclusion/exclusion (against APEASE criteria)
Knowledge	Education	Increasing knowledge or understanding	Included	Considered **affordable**, **practical**, potentially **effective**, potentially **acceptable**, should have limited **side effects** and shouldn’t create significant issues of **equity**
	Training	Imparting skills	Included	Considered **affordable**, **practical**, potentially **effective**, potentially **acceptable**, should have limited **side effects** and shouldn’t create significant issues of **equity**
	Enablement	Increasing means/reducing barriers to increase capability (beyond education/ training) or opportunity (beyond environmental restructuring)	Included	Considered **affordable**, **practical**, potentially **effective**, potentially **acceptable**, should have limited **side effects** and shouldn’t create significant issues of **equity**
Beliefs about consequences	Education	Increasing knowledge or understanding	Included	Considered **affordable**, **practical**, potentially **effective**, potentially **acceptable**, should have limited **side effects** and shouldn’t create significant issues of **equity**
	Persuasion	Using communication to induce positive or negative feelings or stimulate action	Included	Considered **affordable**, **practical**, potentially **effective**, potentially **acceptable**, should have limited **side effects** and shouldn’t create significant issues of **equity**
	Incentivisation	Creating an expectation of reward	Excluded	Not considered **affordable**, unlikely to be **acceptable** to policy makers and would possibly be im**practical** to incentivise over a sustained period of time
	Coercion	Creating an expectation of punishment or cost	Excluded	Not considered **practical** to deliver (as HCPs often want to maintain good and balanced relationships with patients), unlikely to be **acceptable** to HCPs or patients morally and ethically, enforcing punishment or cost onto patients will also likely have unwanted **side effects**, and could reduce **equity** for some sectors of the community
Emotions	Persuasion	Using communication to induce positive or negative feelings or stimulate action	Included	Considered **affordable**, **practical**, potentially **effective**, potentially **acceptable**, should have limited **side effects** and shouldn’t create significant issues of **equity**
	Incentivisation	Creating an expectation of reward	Excluded	Not considered **affordable**, unlikely to be **acceptable** to policy makers and would possible be im**practical** to incentivise over a sustained period of time
	Coercion	Creating an expectation of punishment or cost	Excluded	Not considered **practical** to deliver (as HCPs often want to maintain good and balanced relationships with patients), unlikely to be **acceptable** to HCPs or patients morally and ethically, enforcing punishment or cost onto patients will also likely have unwanted **side effects**, and could reduce **equity** for some sectors of the community
	Restriction	Using rules to reduce the opportunity to engage in the target behaviour (or to increase target behaviour by reducing the opportunity to engage in the competing behaviour)	Excluded	Not considered **practical** to deliver as medicine taking can be carried out alone and so there will be no one present to enforce rules, unlikely to be **acceptable** to patients, HCPs or policy makers as rules often require legislation changes to be enforceable and acted upon
	Environmental Restructuring	Changing the physical or social context	Included	Considered **affordable**, **practical**, potentially **effective**, potentially **acceptable**, should have limited **side effects** and shouldn’t create significant issues of **equity unless** patients do not have access to similar healthcare services or possess similar abilities
	Modelling	Providing an example for people to aspire to or imitate	Excluded	Not considered to be **practical** to deliver as patients do not always have contact with HCPs or other patients when collecting prescriptions for medications, could potentially create disparities in **equity**
	Enablement	Increasing means/reducing barriers to increase capability (beyond education/ training) or opportunity (beyond environmental restructuring)	Included	Considered **affordable**, **practical**, potentially **effective**, potentially **acceptable**, should have limited **side effects** and shouldn’t create significant issues of **equity**

*APEASE* Affordability, practicality, effectiveness/cost-effectiveness, acceptability, side effects, equity, *HCPs* Healthcare Professionals, *TDF* Theoretical Domains Framework

^a^Definitions from Michie, S., L. Atkins, and R. West, *The Behaviour Change Wheel: A Guide to Designing Interventions*. 2014, UK: Silverback Publishing

**Table 2:** Identification of the potential policy categories appropriate for the intervention based on selected intervention functions

Intervention Function	Policy Category	Policy Category Definition^a^	Included/excluded from next stage	Reasons for Inclusion/exclusion (against APEASE criteria)
Education	Communication/Marketing	Using print, electronic, telephonic or broadcast media	Included	Considered **affordable**, **practical**, potentially **effective**, potentially **acceptable**, should have limited **side effects** and shouldn’t create significant issues of **equity**
	Guidelines	Creating documents that recommend or mandate practice. This includes all changes to service provision	Included	Considered **affordable**, **practical**, potentially **effective**, potentially **acceptable**, should have limited **side effects** and shouldn’t create significant issues of **equity**
	Regulation	Establishing rules or principles of behaviour or practice	Excluded	Not considered **practical** for this project as the timeline would not allow for the process of changes to current health practice regulations
	Legislation	Making or changing laws	Excluded	Not considered **practical** for this project as the timeline would not allow for the process of changes to law
	Service Provision	Delivering a service	Included	Considered **affordable**, **practical**, potentially **effective**, potentially **acceptable**, should have limited **side effects** and shouldn’t create significant issues of **equity**
Persuasion	Communication/Marketing	Using print, electronic, telephonic or broadcast media	Included	Considered **affordable**, **practical**, potentially **effective**, potentially **acceptable**, should have limited **side effects** and shouldn’t create significant issues of **equity**
	Guidelines	Creating documents that recommend or mandate practice. This includes all changes to service provision	Included	Considered **affordable**, **practical**, potentially **effective**, potentially **acceptable**, should have limited **side effects** and shouldn’t create significant issues of **equity**
	Regulation	Establishing rules or principles of behaviour or practice	Excluded	Not considered **practical** for this project as the timeline would not allow for the process of changes to current health practice regulations
	Legislation	Making or changing laws	Excluded	Not considered **practical** for this project as the timeline would not allow for the process of changes to law
	Service Provision	Delivering a service	Included	Considered **affordable**, **practical**, potentially **effective**, potentially **acceptable**, should have limited **side effects** and shouldn’t create significant issues of **equity**
Training	Guidelines	Creating documents that recommend or mandate practice. This includes all changes to service provision	Included	Considered **affordable**, **practical**, potentially **effective**, potentially **acceptable**, should have limited **side effects** and shouldn’t create significant issues of **equity**
	Fiscal Measures	Using the tax system to reduce or increase the financial cost	Excluded	Not considered **practical** (partially due to free prescriptions over the age of 60 in UK, which encompasses a large proportion of stroke survivors), unlikely to be **acceptable** to policy makers who would probably need to instigate legislation changes, potentially not **affordable** contingent on the economic climate at the time of the change
	Regulation	Establishing rules or principles of behaviour or practice	Excluded	Not considered **practical** for this project as the timeline would not allow for the process of changes to current health practice regulations
	Legislation	Making or changing laws	Excluded	Not considered **practical** for this project as the timeline would not allow for the process of changes to law
	Service Provision	Delivering a service	Included	Considered **affordable**, **practical**, potentially **effective**, potentially **acceptable**, should have limited **side effects** and shouldn’t create significant issues of **equity**
Environmental Restructuring	Guidelines	Creating documents that recommend or mandate practice. This includes all changes to service provision	Included	Considered **affordable**, **practical**, potentially **effective**, potentially **acceptable**, should have limited **side effects** and shouldn’t create significant issues of **equity**
	Fiscal Measures	Using the tax system to reduce or increase the financial cost	Excluded	Not considered **practical** (partially due to free prescriptions over the age of 60 in UK, which encompasses a large proportion of stroke survivors), unlikely to be **acceptable** to policy makers who would probably need to instigate legislation changes, potentially not **affordable** contingent on the economic climate at the time of the change
	Regulation	Establishing rules or principles of behaviour or practice	Excluded	Not considered **practical** for this project as the timeline would not allow for the process of changes to current health practice regulations
	Legislation	Making or changing laws	Excluded	Not considered **practical** for this project as the timeline would not allow for the process of changes to law
	Environmental/ Social Planning	Designing and/or controlling the physical or social environment	Included	Considered **affordable**, **practical**, potentially **effective**, potentially **acceptable**, should have limited **side effects** and shouldn’t create significant issues of **equity**
Enablement	Guidelines	Creating documents that recommend or mandate practice. This includes all changes to service provision	Included	Considered **affordable**, **practical**, potentially **effective**, potentially **acceptable**, should have limited **side effects** and shouldn’t create significant issues of **equity**
	Fiscal Measures	Using the tax system to reduce or increase the financial cost	Excluded	Not considered **practical** (partially due to free prescriptions over the age of 60 in UK, which encompasses a large proportion of stroke survivors), unlikely to be **acceptable** to policy makers who would probably need to instigate legislation changes, potentially not **affordable** contingent on the economic climate at the time of the change
	Regulation	Establishing rules or principles of behaviour or practice	Excluded	Not considered **practical** for this project as the timeline would not allow for the process of changes to current health practice regulations
	Legislation	Making or changing laws	Excluded	Not considered **practical** for this project as the timeline would not allow for the process of changes to law
	Environmental/ Social Planning	Designing and/or controlling the physical or social environment	Included	Considered **affordable**, **practical**, potentially **effective**, potentially **acceptable**, should have limited **side effects** and shouldn’t create significant issues of **equity**
	Service Provision	Delivering a service	Included	Considered **affordable**, **practical**, potentially **effective**, potentially **acceptable**, should have limited **side effects** and shouldn’t create significant issues of **equity**

*APEASE* Affordability, practicality, effectiveness/cost-effectiveness, acceptability, side effects, equity, *UK* United Kingdom

^a^Definitions from Michie, S., L. Atkins, and R. West, *The Behaviour Change Wheel: A Guide to Designing Interventions*. 2014, UK: Silverback Publishing

The use of APEASE criteria [25], along with consideration of the intervention context, assisted in narrowing down the potentially appropriate intervention functions. Likewise, the same process was used to narrow down the potentially appropriate policy categories. Within Tables 1 and 2, reasons for inclusion/exclusion of the intervention functions and policy categories are presented and examples for exclusion of intervention functions and policy categories are discussed below.

Four intervention functions were not considered to be appropriate for this intervention design (Incentivisation; Coercion; Restriction; and Modelling). For example, Restriction i.e. “use rules to reduce the opportunity to engage in the behaviour” [25], when considering APEASE would likely not be ***acceptable*** or ***practical****.* Medicine taking is often done alone and so there will be no one there to enforce the rules or witness rule breaking, which in turn will limit the ***effectiveness*** of this intervention function. Coercion i.e. “Create an expectation of punishment or cost” [25] seems inappropriate, within the service the intervention would likely be implemented (the NHS), morally and ethically (particularly as this conflicts with HCP ethical frameworks). It is inappropriate to create an expectation of punishment if patients do not take their medicines, given that patients can have valid reasons for not wanting to adhere to regimens such as wanting to stop side effects. Moreover, in terms of APEASE, it is likely not ***acceptable*** to coerce medication adherence, and there will likely be unwanted ***side effects*** from the use of this intervention function, potentially causing the undesired consequence of even worse adherence from patients. Likewise, Incentivisation i.e. “create an expectation of reward” [25], has similar ethical constraints, in addition to potential financial constraints. Although there is emerging evidence (both from systematic review and primary studies) to suggest that incentivisation could have utility in interventions targeting medication adherence [29–33], these studies have predominantly been conducted within the US and have shown promising but varying effect sizes that are not consistently statistically significant. Within the UK NHS context, financial and economic constraints are such that interventions based on financial incentives are unlikely to be adopted. Finally Modelling i.e. “provide an example of people to aspire to or emulate” [25] was felt to limit some ***practicality*** and possibly some ***equity***. The ability to emulate another’s behaviour could be contingent on multiple other factors, such as access to similar healthcare services and patients possessing similar abilities (cognitive, social, physical) to successfully take their medicines in the same manner.

Two policy categories were not considered further for this intervention design (Fiscal Measures and Legislation). For example, Fiscal Measures i.e. “the use of the tax system to reduce or increase the financial cost” [25] is not a ***practical*** policy category to consider. This is partly due to the fact that within the UK, residents over the age of 60 years (a category that a large proportion of stroke survivors come under) do not pay for prescriptions and as such an amendment to taxation systems seems a less ***practical*** option. Furthermore, individuals considered to be living on low incomes or receiving certain types of welfare benefits (such as those unable to work due to illness) can apply for exemptions of paying for prescriptions. In the UK, prescription costs are also comparatively low in comparison to other healthcare systems such as those in the US (£8.80 per item, which equates to US $11.85 or €10.20), and for individuals requiring multiple prescriptions regularly, there are schemes in place to reduce the maximum annual cost to £104. Fiscal measures would likely require legislation changes, something that would rely upon elected politicians’ willingness to propose such changes. There would also be questions of ***affordability*** dependant on the economic climate at the time of the intervention, and thus the use of this policy category could become less ***acceptable***. Legislation i.e. “making or changing laws” [25] was not ***practical*** to focus on within this project as the process involved would be out of scope for a research study.

### Stage 3: Identify content and implementation options

Table 3 displays the process of systematically using an evidence base to select potential BCTs for this intervention. Careful linking of evidence base and theory, underpinned by the BCW [23, 25] and previous work by experts in the field [27, 28] has resulted in a potential 21 BCTs that might be considered for this intervention. Application of APEASE, as well as identifying existing effectiveness of the BCTs within other, similar interventions has enabled the selection to be narrowed down to 11 BCTs (*information about health consequences (5.1)*; *self-monitoring of behaviour (2.3)*; *biofeedback (2.6)*; *information about antecedents (4.3); credible source (9:1)*; *self-monitoring of outcome(s) of behaviour (2.4)*; *pros and cons (9.2)*; *prompts/cues (7:1*); *action planning (1:4)*; *habit formation (8:3)*; *social support (emotional) (3.3)*). Table 4 presents all 21 BCTs, separated into BCTs that will be included or excluded from the next stage of this intervention development. Reasons for inclusion/exclusion of each BCT are summarised in Table 4, assessed against APEASE criteria.

**Table 3:** Identification of the possible BCTs that could be used in the intervention

TDF domains	Intervention Functions Identified	BCTs identified
Knowledge	Education Training Enablement	1. Feedback on outcome(s) of the behaviour (2.7) 2. Provide normative information about others behaviour/ experiences (BM5) 3. Information about health consequences (5.1) 4. Self-monitoring of behaviour (2.3) 5. Provide reassurance (RC10) 6. Feedback on behaviour (2.2) 7. Biofeedback (2.6) 8. Information about antecedents (4.2)
Beliefs about consequences	Education Persuasion	1. Information about health consequences (5:1) 2. Credible source (9:1) 3. Self-monitoring of outcome(s) of behaviour (2.4) 4. Information about antecedents (4.2) 5. Pros and cons (9.2) 6. Salience of consequences (5.2) 7. Information about social and environmental consequences (5.3) 8. Information about emotional consequences (5.6) 9. Anticipated regret (5.5) 10. Comparative imagining of future outcomes (9.3)
Emotions	Persuasion Environmental restructuring Enablement	1. Information about health consequences (5.1) 2. Credible source (9.1) 3. Prompts/cues (7.1) 4. Action planning (1.4) 5. Habit formation (8.3) 6. Self-monitoring of behaviour (2.3) 7. Feedback on behaviour (2.2) 8. Feedback on outcome(s) of the behaviour (2.7) 9. Social support (emotional) (3.3) 10. Reduce negative emotions (11.2)

*BCT* Behaviour Change Technique, *BM5* BCT code relating to a specific focus on the target behaviour (B) and maximising motivation (M), *TDF* Theoretical Domains Framework

**Table 4:** List of included/excluded BCTs with reasons for inclusion/exclusion

BCTs	Reasons for Inclusion/exclusion (against APEASE criteria)
BCTs Included	
Information about health consequences (5.1)	Considered **affordable**, **practical**, potentially **effective**, potentially **acceptable** (for patients and HCPs), should have limited **side effects**
Self-monitoring of behaviour (2.3)	Considered **affordable**, **practical**, potentially **effective**, potentially **acceptable** (for patients and HCPs), should have limited **side effects** and shouldn’t create significant issues of **equity**
Biofeedback (2.6)	Considered **affordable** (as patients’ blood pressure and cholesterol, for example, are often monitored and discussed within routine care), **practical**, potentially **effective** and potentially **acceptable** (for patients and HCPs)
Information about antecedents (4.3)	Considered **affordable**, **practical**, potentially **effective**, potentially **acceptable** (for patients and HCPs), should have limited **side effects** and shouldn’t create significant issues of **equity**
Credible source (9:1)	Considered **affordable**, **practical**, potentially **effective**, potentially **acceptable** (for patients and HCPs), should have limited **side effects** and shouldn’t create significant issues of **equity**
Self-monitoring of outcome(s) of behaviour (2.4)	Considered **affordable** (as patients can access, for example, blood pressure monitors for free from local GP surgeries and pharmacists), **practical**, potentially **effective** and potentially **acceptable** (for patients and HCPs)
Pros and cons (9.2)	Considered **affordable**, **practical**, potentially **effective** and potentially **acceptable** (for patients and HCPs)
Prompts/cues (7:1)	Considered **affordable**, **practical**, potentially **effective**, potentially **acceptable** (for patients and HCPs), should have limited **side effects** and shouldn’t create significant issues of **equity**
Action planning (1:4)	Considered **affordable**, **practical**, potentially **effective**, potentially **acceptable** (for patients and HCPs), should have limited **side effects** and shouldn’t create significant issues of **equity**
Habit formation (8:3)	Considered **affordable**, **practical**, potentially **effective**, potentially **acceptable** (for patients and HCPs), should have limited **side effects** and shouldn’t create significant issues of **equity**
Social support (emotional) (3.3)	Considered **affordable** (as patients may be able to get this support from their own social networks or from community stroke support groups already running), **practical**, potentially **effective**, potentially **acceptable** (for patients and HCPs), should have limited **side effects**
BCTs Excluded	
Feedback on outcome(s) of the behaviour (2.7)	Not considered **practical** as most feedback on behavioural outcomes (for stroke medication adherence) routinely provided in NHS is a form of biofeedback and so would add additional workload if HCPs were providing feedback
Feedback on behaviour (2.2)	Not considered **practical** in this context. Although HCPs based in primary care/community pharmacy have access to prescription acquisition records, this is a proxy measure of adherence and so could be difficult to provide accurate estimates of adherence. Even if stroke survivors provided self-reported accounts of adherence to HCPs/carers and adherence rates were fed back this could be too onerous as an intervention strategy
Provide normative information about others behaviour/ experiences (BM5)	Not considered **practical** to deliver. BCT originally utilised for smoking cessation. The impact of stroke and varying medication regimens will make generalised comparisons challenging
Salience of consequences (5.2)	Not considered **practica**l for negative consequences of non-adherence e.g. use images of the consequences of stroke - hard to demonstrate paralysis, aphasia and other stroke implications in an image. Potential to have unwanted **side effects** also, if BCT evokes upsetting emotional responses. Enhancing view about positive consequences of adherence may not be considered **acceptable** as patient could have suffered a stroke even when adherent to medication and may not find the salience of the consequences meaningful
Information about social and environmental consequences (5.3)	Not considered **acceptable**. Potential ethical issues. E.g. informing patients that it’s not socially acceptable to miss medication doses – patients can have valid reasons for not taking medications
Information about emotional consequences (5.6)	Not considered **acceptable** and could cause unwanted **side effects** if information about negative emotions is given. Provision of this information could be upsetting, and patients can have valid reasons for not taking medications so seems inappropriate in this context. Even if provide information about positive emotions (e.g. taking medicines give peace of mind) may not be considered **acceptable** to those who have suffered a stroke even when adherent to medication and so may not find the information meaningful
Anticipated regret (5.5)	Not considered **acceptable** and could cause unwanted **side effects**. Provision of this information could be upsetting, and patients can have valid reasons for not taking medications so seems inappropriate in this context. If a patient suffered a stroke following good adherence to medications BCT could be considered un**acceptable** by intervention facilitators delivering this BCT
Comparative imagining of future outcomes (9.3)	Not considered **acceptable**. Asking people to imagine different outcomes might not be something HCPs are confident doing or patients are familiar with
Reduce negative emotions (11.2)	Not considered **practical** and potentially not **affordable**. Not all patients would require this type of BCT and training in stress management, for example, would be costly and time/labour intensive
Provide reassurance (RC10)	Not considered **practical**. Patients experiences (e.g. of side effects) are likely not time limited and may vary person to person. Reassurance may not always be the appropriate response and so may not be considered **acceptable**

*APEASE* Affordability, practicality, effectiveness/cost-effectiveness, acceptability, side effects, equity, *BCT* Behaviour Change Technique, *BCTTV1* Behaviour Change Technique Taxonomy Version 1, *HCPs* Healthcare Professionals

## Discussion

This study serves to add to existing stroke and medication adherence literature by presenting an intervention developed using the BCW, underpinned by the TDF, and designed in consideration of implementation into the NHS. Eleven BCTs were identified as potential components for his intervention. BCTs identified included *habit formation (8.4)*, *action planning (1.4)* and *information about health consequences (5.1)*.

Consideration of the intervention context (e.g. time and financial pressures within the NHS), facilitated by using APEASE evaluative criteria [25], has enhanced this process and enabled the development of a focused intervention. This process was important as there is currently a lack of experimental evidence in the literature looking at the effectiveness of particular BCTs targeting specific psychological determinants of medication adherence of stroke survivors. Moreover, inconsistencies in descriptions of published interventions means that it is difficult to establish which BCTs are more effective at targeting medication adherence in stroke survivors. For example, it is often difficult to identify the type of information provision given to participants in an intervention, as varying terms have been used for this intervention component e.g. “an educational booklet” [34] or “reinforcing relevant knowledge on the chronic diseases they are suffering from” [35]. With the development of checklists to support reporting of interventions, such as the Template for Intervention Description and Replication (TIDieR) checklist [36], clear and transparent recording of what is included within interventions may be enhanced.

An understanding of underlying modifiable factors is required in order to change behaviour (e.g. [37]). To achieve this, a systematic review was used in this study that identified potential determinants of medication adherence in stroke survivors to target with intervention [26]. The shortlisted selection of BCTs was generated through systematic linking of determinants to intervention functions, policy categories and associated BCTs, ensuring that the intervention remains evidence-based, theory driven and targets the known modifiable psychological influences on medication adherence in stroke survivors. This will facilitate evaluation of the interventions’ effectiveness when feasibility and pilot testing is carried out, as identification of core intervention components will be possible, something highlighted as important in behaviour change intervention development and reporting [38].

This approach to intervention development (use of TDF, BCW and APEASE) has focused the identification of intervention functions through which BCTs will be delivered (Education, Persuasion, Training, Environmental Restructuring and Enablement). It has also identified the most likely effective BCTs that could be delivered in intervention (for example, *information about health consequences***,** use of a *credible source*, *self-monitoring of the behaviour***,** *social support (emotional)***,** identifying the *pros and cons* to taking medications and using *habit formation***)**. The effectiveness of these BCTs has been supported in previous research discussed below. A Cochrane review, focused on effectiveness of medication adherence interventions, reported that *information***,** *reminders* and *self-monitoring* were included in almost all interventions that showed good effect for improvement in adherence [39]. In 2016, Conn and colleagues [40] conducted a meta-analysis, assessing blood pressure outcomes for medication adherence interventions among adults with hypertension. It was found that BCTs focused on *habit formation* were effective at improving diastolic blood pressure (habit d = 0.477; no habit d = 0.181; *p* < .001) [39]. Moreover, O’Carroll and colleagues [41] piloted a randomised controlled trial (RCT), testing an intervention (incorporating components to support habit formation) targeting adherence to antihypertensive medications in stroke survivors. Significant results were reported, with 10% more doses taken on schedule in the intervention group (intervention, 97%; control, 87%; [95% CI for difference 0.2,16.2]; *p* = 0.048), encompassing BCTs such as *action planning* [41].

This study highlights a key methodological challenge of applying the TDF and BCW in intervention design: selection of suitable BCTs to target underlying theoretical domains was less systematic than identification of intervention functions and policy categories. However, work in 2015 by Cane and colleagues [28] and previous mapping working in 2008 by Michie and colleagues [27] assisted in this process, giving some support and guidance as to which BCTs are likely to target underlying theoretical domains. The use of this literature, along with wider reading of BCTs that are better suited to certain intervention functions and consideration of BCTs that have been previously reported to show reasonable effect in interventions targeting similar behaviours, has been useful in guiding BCT selection.

### Strengths and limitations

#### Strengths

A key strength of this work was the application of the APEASE evaluative criteria to refine selection of intervention functions, policy categories and BCTs. The multiple dimensions covered by the tool lead to a more careful consideration of the realities of implementing this intervention in the NHS context. For example, the APEASE criteria remind intervention designers to consider not only potential acceptability but also potential side effects and the equity implications of a new intervention. As advocated in the literature (e.g. [15, 23]), the present study also demonstrates a transparent and explicit approach to intervention development. In addition, the research team came from multidisciplinary backgrounds (primary care; health psychology) and two of the three authors were also members of an internationally-recognised applied stroke care research group, factors which enabled a more holistic decision making process when attempting to narrow down selection of BCTs. Input from the health psychologists, who have advanced training on the TDF and intervention development applying the BCW facilitated effective use of theory to underpin intervention design.

The generalisability of this study warrants discussion. The overarching method for medication adherence intervention development applied here is generalisable across patient populations. The systematic review to identify psychological influences on stroke survivors’ adherence included papers from any country and so provides an internationally applicable view of the factors that medication adherence interventions for stroke survivors should target. The ability of particular BCTs to change psychological influences is considered to be generic across contexts, in the absence of evidence to the contrary. Therefore, the initial selection of intervention components that could alter psychological determinants of medication adherence is generalisable across stroke survivors in different contexts. However, the application of the APEASE criteria to narrow the choice of potential intervention components requires one to consider both a specific patient population and context. By explicitly stating our judgements of each intervention component, such as a BCT, vs. the APEASE criteria for stroke survivors within the NHS, we enable others to judge the extent to which our final BCT selection would generalise to interventions for stroke survivors in other countries and contexts.

#### Limitations

One limitation of this research may be that assessment of intervention components, such as BCTs, using APEASE criteria involves a certain amount of subjectivity. However, the assessment of potential intervention components were carried out by a multidisciplinary team who have considerable experience and knowledge of adherence interventions and the current healthcare system as it relates to stroke survivors within the UK.

Stroke survivors are a highly diverse patient group, with varying impairments as a result of their strokes, and often significant comorbidities. Our intervention is intended to support stroke survivors who live in the community with some degree of independence, rather than those in institutional care or who are highly dependent on domiciliary carers. Some stroke survivors may have considerable dysphasia. Our intervention is unlikely to entirely meet their needs, and instead, targeted adherence support from doctors, nurses or pharmacists, tailored to the individual’s particular communication difficulties (e.g. expressive or receptive), may be required.

A further potential limitation, and subsequent avenue for future research is the applied definition of adherence the authors have used. There are varying terms used interchangeably to describe a multitude of medication use behaviours including concordance, persistence and adherence. Research efforts have recently provided a more testable and analysable definition [42], that operationalises adherence into three quantifiable stages: initiation, implementation and discontinuation [42]. Our research focuses on the implementation stage, as many stroke survivors initiated some of their medications (e.g. antihypertensives) prior to their stroke. However, in the future, interventions could be designed to have components tailored to target stroke survivors in the three different phases of medication adherence. Most recently, a new metric for medication adherence measurement has been proposed that allows these aspects of behaviour to be assessed by one measure [43]. However, this metric was derived from a sample of patients using inhalers and considers nonadherence related issues such as inhaler technique. Technique may be less relevant medications prescribed for stroke risk factor control and as such requires further research in this population. Enhancing assessment of adherence will strengthen evaluations of intervention effectiveness.

Work such as the establishment of the links between theoretical domains of the TDF and BCTs is relatively new, and is based on hypothesised links and expert consensus. Therefore, more work is required to provide empirical, experimental evidence showing that behaviour change is possible through delivery of specific BCTs targeting underlying determinants known to influence the behaviour. However, careful exploration of the literature to identify BCTs, alongside pragmatic decision making from a multidisciplinary team regarding suitable BCTs based on evaluative criteria, should enable a more realistic and theory-driven approach.

#### Future research

The next stage of this project will be to explore the acceptability of the proposed intervention components, as well as potential modes of delivery (face-to-face, phone, text, email, website etc.) with key stakeholders (healthcare professionals (HCPs)and stroke survivors). Exploration of the most acceptable way to operationalise the BCTs and the acceptability of the overall BCT will be undertaken. For example, the BCT Action planning will be considered. This BCT could be operationalised as asking a participant to make a plan about exactly when and where they will take their first daily dose of medication, and interviews will explore the acceptability and utility of this. Consideration of how this BCT should be delivered (e.g. in a face-to-face discussion with a HCP, developed over the phone with a HCP, through development of the plans prompted via email) will also be explored in this study.

## Conclusions

Use of the BCW and application of the APEASE criteria, to assess the intervention development in context (the NHS) has enabled a novel and practical intervention to be developed, targeting medication adherence in stroke survivors. Previous research identified three key TDF domains to underpin this intervention design (‘Beliefs about consequences’, ‘Knowledge’ and ‘Emotions’). APEASE criteria supported refinement of potential intervention components. Five (5/9) intervention functions and five (5/7) policy categories were identified as possible intervention options. Eleven BCTs, from an initial list of 21, including *habit formation*, *information about health consequences* and *prompts/cues* were considered potentially effective. Feasibility testing is now underway to explore the perceived acceptability of the potential intervention components, together with perceived optimal modes of intervention delivery, through semi-structured interviews with stroke patients and healthcare professionals.

## Abbreviations

APEASE: Affordability practicality effectiveness/cost-effectiveness acceptability side effects equity
BCT: Behaviour Change Technique
BCTTV1: Behaviour Change Technique Taxonomy Version 1
BCW: Behaviour Change Wheel
GP: General practitioner
HCP: Healthcare professional
MRC: Medical Research Council
MUR: Medication Use Review
NHS: National Health Service
RCT: Randomised controlled trial
TDF: Theoretical Domains Framework
TIDieR: Template for intervention description and replication
UK: United Kingdom

## References

[1] Young J, Forster A (2007). Review of stroke rehabilitation. BMJ.

[2] Feigin VL, Norrving B, Mensah GA (2017). Global burden of stroke. Circ Res.

[3] National Collaborating Centre for Chronic C. National Institute for Health and Clinical Excellence: Guidance (2008). Stroke: National Clinical Guideline for diagnosis and initial Management of Acute Stroke and Transient Ischaemic Attack (TIA).

[4] Mohan KM, Wolfe CDA, Rudd AG, Heuschmann PU, Kolominsky-Rabas PL, Grieve AP (2011). Risk and cumulative risk of stroke recurrence: a systematic review and meta-analysis. Stroke.

[5] Department of Health DH (2007). National Stroke Strategy.

[6] Nunes V, Neilson J, O’Flynn N, Calvert N, Kuntze S, Smithson H (2009). Clinical guidelines and evidence review for medicines adherence: involving patients in decisions about prescribed medicines and supporting adherence.

[7] Sappok T, Faulstich A, Stuckert E, Kruck H, Marx P, Koennecke HC (2001). Compliance with secondary prevention of ischemic stroke: a prospective evaluation. Stroke.

[8] Yusuf S (2002). Two decades of progress in preventing vascular disease. Lancet.

[9] Sacco RL, Adams R, Albers G, Alberts MJ, Benavente O, Furie K (2006). Guidelines for prevention of stroke in patients with ischemic stroke or transient ischemic attack: a statement for healthcare professionals from the American Heart Association/American Stroke Association Council on stroke: co-sponsored by the council on cardiovascular radiology and intervention: the American Academy of Neurology affirms the value of this guideline. Stroke.

[10] Warlow CP, Van Gijn J, Dennis MS, Wardlaw JM, Bamford JM, Hankey GJ (2008). Stroke: practical management.

[11] Al AlShaikh S, Quinn T, Dunn W, Walters M, Dawson J (2016). Predictive factors of non-adherence to secondary preventative medication after stroke or transient Ischaemic attack: a systematic review and meta-analyses. Eur Stroke J.

[12] Lager KE, Mistri AK, Khunti K, Haunton VJ, Sett AK, Wilson AD. Interventions for Improving Modifiable Risk Factor Control in the Secondary Prevention of Stroke. Cochrane Database of Syst Rev. 2014;(5)2:Cd009103. 10.1002/14651858.CD009103.pub2. 10.1002/14651858.CD009103.pub2 24789063

[13] Wessol JL, Russell CL, Cheng AL (2017). A systematic review of randomized controlled trials of medication adherence interventions in adult stroke survivors. J Neurosci Nurs.

[14] Nieuwlaat R, Wilczynski N, Navarro T, Hobson N, Jeffery R, Keepanasseril A, et al. Interventions for enhancing medication adherence. Cochrane Database Syst Rev. 2014;(11):CD000011. 10.1002/14651858.CD000011. 10.1002/14651858.CD000011.pub4 PMC7263418 25412402

[15] Craig P, Dieppe P, Macintyre S, Michie S, Nazareth I, Petticrew M. Developing and evaluating complex interventions: the new Medical Research Council guidance. BMJ. 2008;337:a1655. 10.1136/bmj.a1655 PMC2769032 18824488

[16] Bartholomew LK, Parcel GS, Kok G (1998). Intervention mapping: a process for developing theory- and evidence-based health education programs. Health Educ Behav.

[17] Kok G, Gottlieb NH, Peters GJ, Mullen PD, Parcel GS, Ruiter RA (2016). A taxonomy of behaviour change methods: an intervention mapping approach. Health Psychol Rev.

[18] Michie S, Richardson M, Johnston M, Abraham C, Francis J, Hardeman W (2013). The behavior change technique taxonomy (v1) of 93 hierarchically clustered techniques: building an international consensus for the reporting of behavior change interventions. Ann Behav Med.

[19] Connor M, Norman P (2015). Predicting and changing health behaviour: research and practice with social cognition models.

[20] Michie S, West R, Campbell R, Brown J, Gainforth H (2014). ABC of behaviour change theories.

[21] Michie S, Johnston M, Abraham C, Lawton R, Parker D, Walker A (2005). Making psychological theory useful for implementing evidence based practice: a consensus approach. Qual Saf Health Care.

[22] Cane J, O’Connor D, Michie S (2012). Validation of the theoretical domains framework for use in behaviour change and implementation research. Implement Sci.

[23] Michie S, van Stralen MM, West R (2011). The behaviour change wheel: a new method for Characterising and designing behaviour change interventions. Implement Sci.

[24] Bailey Julia V, Webster Rosie, Hunter Rachael, Griffin Mark, Freemantle Nicholas, Rait Greta, Estcourt Claudia, Michie Susan, Anderson Jane, Stephenson Judith, Gerressu Makeda, Ang Chee Siang, Murray Elizabeth (2016). The Men’s Safer Sex project: intervention development and feasibility randomised controlled trial of an interactive digital intervention to increase condom use in men. Health Technology Assessment.

[25] Michie S, Atkins L, West R (2014). The behaviour change wheel: a guide to designing interventions.

[26] Crayton Elise, Fahey Marion, Ashworth Mark, Besser Sarah Jane, Weinman John, Wright Alison J. (2017). Psychological Determinants of Medication Adherence in Stroke Survivors: a Systematic Review of Observational Studies. Annals of Behavioral Medicine.

[27] Michie S, Johnston M, Francis J, Hardeman W, Eccles M (2008). From theory to intervention: mapping theoretically derived Behavioural determinants to behaviour change techniques. Appl Psychol.

[28] Cane J, Richardson M, Johnston M, Ladna R, Michie S (2015). From lists of behaviour change techniques (BCTs) to structured hierarchies: comparison of two methods of developing a hierarchy of BCTs. Br J Health Psychol.

[29] DeFulio A, Silverman K (2012). The use of incentives to reinforce medication adherence. Prev Med.

[30] Garza KB, Owensby JK, Braxton Lloyd K, Wood EA, Hansen RA (2016). Pilot study to test the effectiveness of different financial incentives to improve medication adherence. Ann Pharmacother.

[31] Priebe S., Yeeles K., Bremner S., Lauber C., Eldridge S., Ashby D., David A. S., O'Connell N., Forrest A., Burns T. (2013). Effectiveness of financial incentives to improve adherence to maintenance treatment with antipsychotics: cluster randomised controlled trial. BMJ.

[32] Priebe S, Bremner SA, Lauber C, Henderson C, Burns T (2016). Financial incentives to improve adherence to antipsychotic maintenance medication in non-adherent patients: a cluster randomised controlled trial. Health Technol Assess.

[33] Volpp KG, Loewenstein G, Troxel AB, Doshi J, Price M, Laskin M (2008). A test of financial incentives to improve warfarin adherence. BMC Health Serv Res.

[34] Rinfret S, Lussier M-T, Peirce A, Duhamel F, Cossette S, Lalonde L (2009). The impact of a multidisciplinary information technology–supported program on blood pressure control in primary care. Circ Cardiovasc Qual Outcomes.

[35] Wong MC, Liu KQ, Wang HH, Lee CL, Kwan MW, Lee KW (2013). Effectiveness of a pharmacist-led drug counseling on enhancing antihypertensive adherence and blood pressure control: a randomized controlled trial. J Clin Pharmacol.

[36] Hoffmann T. C., Glasziou P. P., Boutron I., Milne R., Perera R., Moher D., Altman D. G., Barbour V., Macdonald H., Johnston M., Lamb S. E., Dixon-Woods M., McCulloch P., Wyatt J. C., Chan A.-W., Michie S. (2014). Better reporting of interventions: template for intervention description and replication (TIDieR) checklist and guide. BMJ.

[37] Hardeman W, Sutton S, Griffin S, Johnston M, White A, Wareham NJ (2005). A causal modelling approach to the development of theory-based behaviour change programmes for trial evaluation. Health Educ Res.

[38] Michie S, Fixsen D, Grimshaw JM, Eccles MP (2009). Specifying and reporting complex behaviour change interventions: the need for a scientific method. Implement Sci.

[39] Haynes RB, Ackloo E, Sahota N, McDonald HP, Yao X. Interventions for Enhancing Medication Adherence. Cochrane Database Syst Rev. 2008;(2)16:Cd000011. 10.1002/14651858.CD000011.pub3. 10.1002/14651858.CD000011.pub3 18425859

[40] Conn VS, Ruppar TM, Chase JD (2016). Blood pressure outcomes of medication adherence interventions: systematic review and meta-analysis. J Behav Med.

[41] O’Carroll RE, Chambers JA, Dennis M, Sudlow C, Johnston M (2013). Improving adherence to medication in stroke survivors: a pilot randomised controlled trial. Ann Behav Med.

[42] Vrijens B, De Geest S, Hughes DA, Przemyslaw K, Demonceau J, Ruppar T (2012). A new taxonomy for describing and defining adherence to medications. Br J Clin Pharmacol.

[43] Greene G, Costello RW, Cushen B, Sulaiman I, Mac Hale E, Conroy RM (2018). A novel statistical method for assessing effective adherence to medication and calculating optimal drug dosages. PLoS One.

